# Single Nucleotide Polymorphisms in *PEMT* and *MTHFR* Genes are Associated with Omega 3 and 6 Fatty Acid Levels in the Red Blood Cells of Children with Obesity

**DOI:** 10.3390/nu11112600

**Published:** 2019-10-30

**Authors:** Vlad Serafim, Adela Chirita-Emandi, Nicoleta Andreescu, Diana-Andreea Tiugan, Paul Tutac, Corina Paul, Iulian Velea, Alexandra Mihailescu, Costela Lăcrimioara Șerban, Cristian G. Zimbru, Maria Puiu, Mihai Dinu Niculescu

**Affiliations:** 1 Centre of Genomic Medicine, Genetics Discipline, “Victor Babes” University of Medicine and Pharmacy, Timisoara 300041, Romania; 2 The National Institute of Research and Development for Biological Sciences, Bucharest 060031, Romania; 3 “Louis Turcanu” Clinical Emergency Hospital for Children, Timisoara 300011, Romania; 4 Paediatrics Department, “Victor Babes” University of Medicine and Pharmacy, Timisoara 300041, Romania; 5 2nd Paediatrics Clinic, Clinical Emergency County Hospital, Timisoara 300041, Romania; 6 Department of Functional Sciences, ”Victor Babes” University of Medicine and Pharmacy, Timișoara 300041, Romania; 7 Faculty of Automation and Computer Science, Politehnica University of Timisoara, Timisoara 300223, Romania; 8 Advanced Nutrigenomics, 130 Rainbow Ct, Cary, NC 27511, USA

**Keywords:** polyunsaturated fatty acids, *PEMT*, *MTHFR*, children, obesity, red blood cells

## Abstract

Polyunsaturated fatty acids (PUFAs) play important roles in health and disease. PUFA levels are influenced by nutrition and genetic factors. The relationship between PUFA composition in red blood cells (RBCs) and genetic variations involved in PUFA metabolism has not been investigated in children with obesity. This study evaluated the association between several genetic variations and PUFA levels in RBCs in children with obesity. One hundred ninety-six children with obesity (101 females, 95 males) were evaluated using anthropometric measurements, dietary intakes, plasma and RBC PUFA quantification, blood biochemistry, and 55 single nucleotide polymorphisms within 14 genes. phosphatidylethanolamine *N*-methyltransferase (*PEMT*) rs1109859 and methylenetetrahydrofolate reductase gene (*MTHFR*) rs4846052 genotypes were associated with PUFA levels in RBCs. PUFA intake did not influence the RBC eicosapentaenoic acid (EPA) and docosahexaenoic acid (DHA) levels. Higher RBC DHA and EPA levels were observed for *PEMT* rs1109859 GG and GA genotypes versus the AA genotype. Higher levels of RBC DHA, EPA, arachidonic acid (ARA), and linoleic acid (LA) and were observed for *MTHFR* rs4846052 TT genotype versus TC and CC genotypes. Genetic variations in *PEMT* rs1109859 and *MTHFR* rs4846052 were associated with different PUFA levels in RBC membranes and are estimators for PUFA species in RBCs. Further research is needed to establish whether these genotype-specific alterations are specific to overweight children.

## 1. Introduction

Within the research devoted to obesity, nutrition studies are at the forefront of the efforts aimed at combating this major epidemic. Among these, considerable efforts are involved in finding how the quality, quantity, and types of fats are contributing to both the obesity onset and progression, or to its reduction. This study addresses the potential role of genetic variations in the modulation of polyunsaturated fatty acid (PUFA) levels in children with obesity. PUFA levels are influenced by both nutrition and genetic factors, separately [1,2], or by diet–gene interactions [3]. PUFAs are obtained from diet and synthesized endogenously from their precursor molecules (e.g., linoleic acid for omega-6, and alpha-linolenic acid for omega-3 species). Several genes may influence PUFAs’ status in the body. In PUFAs’ endogenous synthesis, the fatty acid desaturase (FADS) gene cluster (controlling the desaturation) and genes controlling the elongation (ELOVL genes) [4] are involved; variations in these genes impact PUFA levels in humans, including children [5]. Additionally, genetic variations in genes involved in the synthesis of phospholipids and one-carbon metabolism may further impact PUFAs’ status. Phosphatidylcholine (PtdCho) is the most common phospholipid in cell membranes. PtdCho is synthesized through distinct pathways, either through the Cytidine 5’-diphosphocholine (CDP-choline) pathway or by the conversion of phosphatidylethanolamine to PtdCho by phosphatidylethanolamine *N*-methyltransferase (*PEMT*) [6], which influences the abundance of certain PUFAs in phospholipids structure [2]. The methylenetetrahydrofolate reductase gene (*MTHFR*), involved in one-carbon metabolism, may also impact omega-3 PUFA levels [7,8], probably by influencing *S*-Adenosyl methionine synthesis, which in its turn influences PUFAs’ phospholipid composition.

The fatty acid composition of red blood cells (RBCs) reflects the fatty acid composition in other organs [9]. Therefore, the fatty acid levels in RBC membranes could be potentially relevant biomarkers for the evaluation of PUFAs’ status in the human body, and could potentially improve the assessment of PUFA homeostasis, adding relevant information about the roles PUFA might have in relation to obesity-associated metabolic disorders such as dyslipidemia [10], and also reveal other relationships with dietary intakes and genetic variations.

The PUFA status of RBC membranes has been scarcely studied in the context of childhood obesity and genetic variability. Considering the importance of the fatty acid composition in cell membranes, this study focused on the association between several single nucleotide polymorphisms (SNPs) and PUFA content in the RBCs of children with obesity.

## 2. Materials and Methods 

### 2.1. Participants and Samples

Two hundred children (95 males, 105 females) aged 7–18 years, with obesity defined by body mass index (BMI) > +2 SD over the World Health Organization (WHO) 2007 reference [11], were evaluated at the 2nd Pediatric Clinic of Clinical Emergency County Hospital Timisoara, Romania. Exclusion criteria were diagnosis of any type of cancer or medical history of cancer; any psychiatric disorder; blood coagulation disorders; endocrine-induced obesity (Cushing syndrome, hypothyroidism, growth hormone deficit); hypothalamus-induced obesity (Babinski–Fröhlich syndrome); genetic syndromes (Prader–Willi, achondroplasia, Bardet–Biedl, Fanconi, Turner, etc.), and personal history for convulsive disorders, nephrotic syndrome, or asthma with corticoid treatment. Four individuals (females) were excluded due to incomplete assessment data.

Participants and their parents or legal guardians were informed about the aims and methods of the study. Informed consent was obtained verbally from the participating children and in writing from their parents or legal guardians. The study was approved by the Ethics Committee of the “Victor Babes” University of Medicine and Pharmacy (6/20.06.2016), Timisoara, Romania, and conducted in accordance with the Declaration of Helsinki. The study was registered at ClinicalTrials.gov (NCT02837367).

Blood samples were collected after overnight fasting (at least 6 h) in EDTA sterile vacutainers. 

### 2.2. Anthropometric Measurements

Anthropometric measurements were performed in light clothing, without shoes, in the morning, following international guidelines as previously described [12]. Weight and height were measured using an electronic scale with a stadiometer. Measurements for height and weight were recorded to the nearest 0.5 cm and 0.5 kg, respectively. BMI was calculated as kg/m^2^. Standardized BMI-for-age *z*-scores (*z*BMI) were calculated according to the WHO guidelines in order to account for the age and gender of children [11]. 

### 2.3. Food Intake

Food intake was evaluated using 5-pass 24 h dietary recalls as previously described [13]. Briefly, the recalls were administered four times to each participant if older than 13 years of age, or to both a parent and the child if the participant was younger. The declared amounts for each day investigated (foods and drinks) were converted to energy and macro- and micronutrient intakes using a web-application (Nutritio, Bucharest, Romania, https://nutritioapp.com) based on the U.S. Department of Agriculture (USDA) Food and Nutrient Database for Dietary Studies, with appropriate adaptations for local foods. 

### 2.4. Hematological and Biochemical Tests

Complete blood count was assessed using flux cytometry and cytochemistry by ISO 15189-accredited medical laboratory, acting as an external partner. 

Total plasma concentrations of aspartate aminotransferase (AST), alanine aminotransferase (ALT), C reactive protein (CRP), total cholesterol, high-density lipoprotein (HDL) cholesterol, triglycerides, and glucose were performed on an Ortho Clinical Vitros 350 Chemistry System (Ortho Clinical Diagnostics Inc, Raritan, NJ, USA), using its standardized reagents, following the manufacturer’s protocols. Homocysteine and insulin were measured by ELISA method on an Epoch Microplate Spectrophotometer (BioTek Instruments Inc., Winooski, VT, USA). The kits used for insulin were acquired from Fortress Diagnostics (Antrim, United Kingdom), and for homocysteine (Axis Homocysteine Enzyme Immunoassay) from IBL International GMBH, Hamburg, Germany. The analyzer was calibrated and maintained according to the manufacturer’s instructions.

A homeostatic model assessment for insulin resistance (HOMA-IR) was calculated using the following formula: fasting insulin (mIU/L) × fasting glucose (mmol/L)/22.5. 

### 2.5. Fatty Acids Quantification

The blood samples were subjected to centrifugation at 1000× *g* for 10 min at room temperature. After the plasma was transferred into another tube, the RBCs were washed twice with PBS. If not immediately processed, the samples were stored at −80 °C. The samples (plasma and RBCs) were processed and analyzed using a previously described protocol [14]. Three different measurements were obtained for each sample: plasma free fatty acids (FFA), plasma conjugated fatty acids, and RBC membrane fatty acids. The results were expressed in µmol/L. The conjugated fatty acid levels in plasma were estimated by subtracting the FFA plasma levels from total fatty acids levels.

### 2.6. Preparation of Libraries for Next-Generation Sequencing

Genomic DNA was isolated from whole blood using the MagCore^®^ Extractor System and MagCore^®^ Genomic DNA Whole Blood Kit (RBC Bioscience, New Taipei City, Taiwan), following the manufacturer’s protocol. Genotyping was performed on a MiSeq sequencer (Illumina, San Diego, CA, USA) using a custom-made hotspot sequencing kit for 55 SNPs within 14 genes selected as previously being associated with increased lipids, non-alcoholic fatty liver, or cardiovascular disease [15].

Amplicon sequencing libraries were prepared from 20 ng of DNA per sample according to the AmpliSeq protocol (Illumina Inc, San Diego, CA, USA). Libraries were generated with dual indices (19 PCR cycles) followed by normalization and pooling. The pooled libraries were paired-end (2 × 150) sequenced on a micro flow cell with V2 chemistry on a MiSeq instrument (Illumina Inc, San Diego, CA, USA). 

### 2.7. Analysis of Genetic Variants 

After demultiplexing and the generation of FASTQ files, sequence alignment to the reference genome and sequence quality filtering were performed using the Illumina MiSeq Reporter v2.6 platform. The sequences were aligned with Burrows-Wheeler Aligner (BWA)and variant calling was performed with Genome Analysis Toolkit (GATK) using the human reference sequence hg19/GRCh37. Variant calling was performed on the variant call format (VCF) output files by evaluating the coverage (the number of times that a targeted variant is read during the sequencing) and the quality score (*Q*-score; the estimated probability of the base call being wrong). VCF files were further subjected to annotation using ANNOVAR [16] with the dataset dbNSFP 35a [17]. 

### 2.8. Statistical Analyses

Data were analyzed using IBM-SPSS version 25 (IBM, Armonk, New York, USA). The Shapiro–Wilk test was used to determine if the data set had normal distribution. The Levene test was used for assessing homogeneity of variance. The Mann–Whitney U test was used to evaluate the significance of differences between females and males in all variables studied. Correlations were evaluated for statistical significance using the Spearman’s test. 

The Kruskal–Wallis test for independent samples was performed using PUFA measurements in plasma and RBCs (alpha-linolenic acid (ALA), arachidonic acid (ARA), docosahexaenoic acid (DHA), eicosapentaenoic acid (EPA), and linoleic acid (LA)) as the dependent variables, and with the 55 SNPs as predictors. Where the tests gave significant results, the Mann–Whitney U test was used for the supplementary evaluation of differences in PUFA levels between the three genotypes for the SNPs found to be significantly associated with fatty acid measurements. Boxplot graphics were used to display the distribution of DHA, LA, ARA, and EPA between *PEMT* and *MTHFR* genotypes, respectively.

Univariate analyses of variance models were performed for evaluating joint variability of genotypes, gender, hemoglobin, hematocrit, and PUFA dietary intakes.

## 3. Results

Sixty-five percent (128/196) of children in the cohort lived in an urban environment, while the rest lived in rural areas. Descriptive statistics for anthropometric data, biochemical analysis, PUFA measurements in RBC membranes, and dietary intakes of the 196 participants are presented in Table 1. As most of the variables were non-normally distributed, median and interquartile range (IQR) are presented. Significant differences were found between genders for hemoglobin, zBMI, and RBC levels of ALA and LA; all were higher in males. 

PUFA species in RBC membranes did not correlate with total RBC number, hemoglobin, hematocrit, estimated macronutrients, ALA, ARA, DHA, EPA, and LA dietary intakes, nor with zBMI (stratification by gender did not show significance, data not shown). PUFAs from RBCs did not correlate with age, except for EPA (−0.218, *p* = 0.002). Environment (urban versus rural) did not correlate with the PUFA levels. Mann–Whitney U test showed that gender is significantly associated with ALA and LA levels in RBC (*p* values of 0.038 and 0.029, respectively). The zBMI levels positively correlated with AST (0.161, *p* = 0.023), ALT (0.232, *p* = 0.001), and with age (0.441, *p* ≤ 0.001).

Descriptive statistics for the measurement of fatty acids in RBC membranes and plasma are presented as median and IQR in Table A1. DHA in RBC membranes correlated with plasma free DHA (0.236, *p* = 0.001) and with plasma conjugated DHA (0.220, *p* = 0.002). EPA from RBCs correlated with plasma free EPA (0.375, *p* ≤ 0.001) and with plasma conjugated EPA (0.433, *p* ≤ 0.001). ALA, ARA, and LA did not correlate between measurements in plasma and RBC membranes.

**Figure 1:**
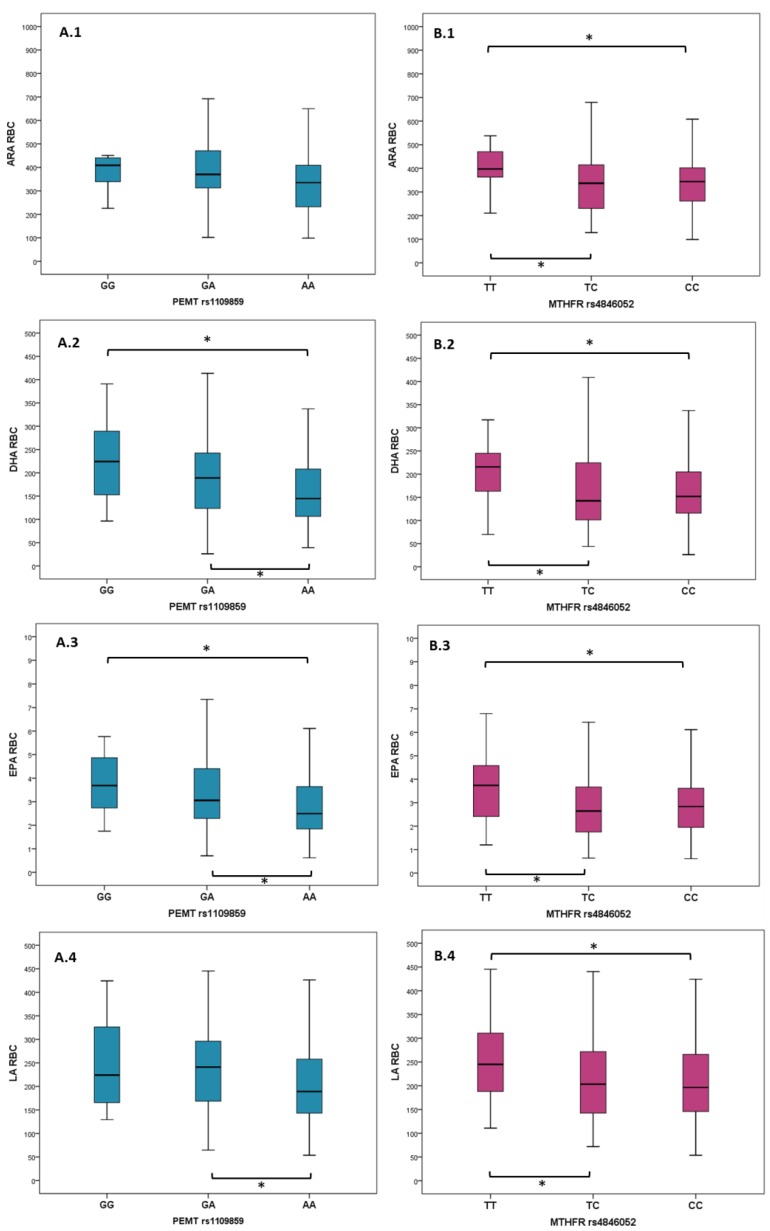
Distribution of PUFA species grouped by *PEMT* rs1109859 (**A**) and *MTHFR* rs4846052 genotypes (**B**). Mann–Whitney U test was used to assess statistical significance between groups. Significant differences are marked with *, and the brackets indicate the two groups for which these differences were identified. Horizontal lines, within each boxplot, indicate minimum, first quartile (Q1), median, third quartile (Q3), and maximum. Legend: ARA—arachidonic acid, DHA—docosahexaenoic acid, EPA—eicosapentaenoic acid, LA—linoleic acid, RBC—red blood cell membrane; *MTHFR*—methylenetetrahydrofolate reductase; *PEMT*—phosphatidylethanolamine *N*-methyltransferase.

Using the independent-samples Kruskal–Wallis test, *PEMT* rs1109859 and *MTHFR* rs4846052 genotypes were found to significantly associate with fatty acid levels (except for ALA) measured in RBC membranes, but not in plasma (data not presented). The rest of the SNPs did not show significant association with RBC PUFAs (frequencies of all 55 SNPs are shown in Table A2). ARA, DHA, EPA, and LA levels in RBCs were significantly different between the patient’s groups identified based on their *PEMT* rs1109859 genotype. ARA, DHA, and EPA levels were significantly different between the patient’s groups identified based on their *MTHFR* rs4846052 genotype (Table 2). Figure 1 displays the distribution of ARA, EPA, DHA, and LA levels between genotypes and differences that are statistically significant between groups (Mann–Whitney U test). The p values are presented in Table A3).

Univariate analysis of variance controlled for the dietary intakes and indicated that DHA, EPA, and LA dietary intakes did not influence the DHA, EPA, and LA levels in RBC membranes. Univariate analysis of variance also indicated that gender influenced ALA and LA levels in RBCs, while hematocrit influenced ARA levels. Complete results for univariate analysis of variance models are shown in Table A4.

## 4. Discussion

This study analyzed omega-6 and omega-3 fatty acid levels in plasma and in the RBC membranes of children with obesity in relation to their food intake and to 55 SNPs from 13 genes associated with lipid metabolism. 

Significant differences were identified between females and males regarding standardized body mass index for age (zBMI), with males presenting a higher degree of obesity. This is a previously described feature of the Romanian population [18,19] and in other countries [20]. However, the zBMI did not suggest an association with PUFAs measured in plasma or RBC membranes. The difference in hemoglobin levels between females and males, lower in females, has been already described in other studies, and it is probably explained by menstrual cycle blood loss [21]. Although this difference is significant, the hemoglobin and hematocrit did not correlate with the PUFAs measured in RBC membranes. However, hematocrit, when added in the model with *PEMT* rs1109859, was found to influence the ARA level in RBCs. The differences between males and females in ALA and LA, measured in RBCs, were not reported previously and need to be re-evaluated in larger studies. 

Aminotransferases directly correlated with zBMI in the present study. This finding was previously reported by others and could be considered as a surrogate marker for nonalcoholic fatty liver disease and associated with metabolic syndrome in children [22]. Additionally, zBMI correlated directly with the age of children in our cohort. In other cohorts, higher adiposity was associated with older age of children in national and international studies [20,23,24]. 

The negative correlation of EPA with age and weight in children was previously shown in another cohort and was associated with response to supplementation, EPA increasing less in those with higher BMIs [25].

The correlations between EPA and DHA measurements from plasma and RBCs in children with obesity have not been previously investigated and need to be re-evaluated in other larger studies that should include control (lean) groups. 

PUFA levels measured in plasma were not associated with any of the investigated genotypes, possibly because the plasma reflects a short-term metabolic status for PUFAs [26]. RBCs, on the other hand, contain fatty acids esterified in phospholipids, which are structural constituents of cell walls, and therefore their fatty acid composition is stable over a longer period, considering that RBCs have a 100-day life-span [27]. 

Although the RBC PUFA levels in the present study were not influenced by estimated intakes, they associated significantly with two SNPs in the *PEMT* and *MTHFR* genes, suggesting that, in children with obesity, genetic variability could be predictive for PUFA composition in RBCs. GG and GA genotypes, when compared to the AA genotype for the *PEMT* rs1109859, were associated with higher levels of DHA and EPA in RBCs. This is in agreement with previous findings [28], suggesting that the PtdCho synthetized via the *PEMT* pathway contains mainly PUFAs (mainly ARA and DHA) while the CDP-choline pathway forms PtdCho containing medium-chain and saturated fatty acids. Another study also found similar evidence, indicating that DHA composition from plasma PtdCho may be a marker for *PEMT* activity [2]. Our results strengthen further the hypothesis that fatty acid composition of PtdCho derived from the *PEMT* pathway is different from that obtained through the CDP–choline pathway. 

PUFA levels in RBCs were also associated with genetic variations for the *MTHFR* gene (rs4846052). To the best of our knowledge, there have been no other previously published reports assessing this association. However, there is evidence that 5-methyltetrahydrofolate (5-mTHF) supplementation can modify the phospholipid fatty acid pattern [29] and can be explained by the fact that 5-mTHF influences methionine bioavailability that is necessary for *S*-adenosylmethionine synthesis. *S*-adenosylmethionine is involved in several cellular transmethylation reactions, including phosphatidylethanolamine methylation [6]. Although the mechanism still needs to be further clarified, our results suggest that the *MTHFR* rs4846052 genotype influences PUFA levels in the RBC membranes, with the TT genotype being associated with higher levels of PUFAs in the RBC membranes compared to that of TC and TT genotypes. However, it is unknown if higher levels of PUFAs in the RBC membranes represent a protective phenotype. 

This study examined, for the first time, the association between *PEMT* and *MTHFR* polymorphisms and fatty acid concentrations in RBC membranes in children with obesity. The study indicated that two SNPs analyzed (located on chromosomes 1 and 17, respectively) could have an important role on the composition of PUFAs in RBCs, possibly due to alterations in PUFA metabolism. Therefore, it is possible that such genetic variations could also contribute to a better understanding of whether PUFA intakes are dependent on such genotypes, and subsequently refine PUFA recommended intakes in children with obesity who may have different requirements as a result of these genetic variations. *PEMT* and *MTHFR* genes contribute, within the methylation pathways, to the regulation of methionine and PUFA homeostasis via the de novo choline synthesis and its incorporation into PtdCho [6]. As the fatty acid composition of PtdCho in cell membranes, especially EPA and DHA distribution, has a protective role against the development of Alzheimer’s disease [30], obesity-induced metabolic disorders, and cardiovascular diseases in mice [31], this remains a significant point for further research. 

The absence of a lean control group is the main limitation of the study. Additionally, this study has not provided a longitudinal analysis for the status of fatty acids in children with different genotypes over time. The assessment of dietary intake using 24 h recalls also has limitations, mostly due to underreporting, as previously shown [32]. However, such recalls have been used widely and represent a validated method for dietary assessment [33,34,35]. Finally, one technical limitation consisted of ARA intakes not being available using the Nutritioapp at the time of this study.

## 5. Conclusions

Genetic variations in *PEMT* (rs1109859) and *MTHFR* (rs4846052) were associated with alterations in the content of PUFA species in RBC membranes. This finding suggests that the genetic status of *PEMT* and *MTHFR* genes may contribute to PUFA homeostasis and, therefore, could contribute to PUFA status in children with obesity. Further research is needed to establish whether these genotype-specific alterations are specific to overweight children. 

## Figures and Tables

**Table 1:** Descriptive statistics for anthropometric data, biochemical analysis, PUFA measurements in RBCs, and dietary intakes. Mann–Whitney U test was used to analyze gender differences (test is significant at *p* value ≤ 0.05).

Variables	All *n* = 196		Females *n* = 101		Males *n* = 95		*p* Value
	Median	IQR	Median	IQR	Median	IQR	
**Anthropometric data**							
Age (years)	12	5	12.00	5.0	12.00	4.75	0.787
*z*BMI	3.13	1.20	2.83	1.21	3.44	1.37	0.001
**Hematological and biochemical analysis**							
Hemoglobin (g/dL)	13.60	1.20	13.50	1.10	13.70	1.47	0.034
Hematocrit (%)	40.20	2.85	39.90	2.80	40.25	3.40	0.152
HOMA-IR	3.95	2.95	4.04	3.04	3.94	3.09	0.470
TC (mg/dL)	174.00	54.00	166.00	52.00	182.50	55.50	0.109
TG (mg/dL)	129.00	86.50	125.00	88.00	133.00	99.75	0.237
HDL (mg/dL)	46.00	18.00	43.00	19.00	48.00	6.75	0.085
AST (U/L)	29.00	14.00	29.00	14.00	29.50	15.50	0.127
ALT (U/L)	32.00	15.00	31.00	16.00	33.50	14.75	0.167
CRP (mg/dL)	4.70	7.90	4.80	8.70	4.75	7.72	0.315
Homocysteine (µmoL/L)	14.78	8.78	14.16	8.08	15.33	9.28	0.398
**PUFA measurements in RBCs**							
ALA RBC (µmol/L)	1.41	1.00	1.28	1.00	1.46	1.1	0.038
ARA RBC (µmol/L)	354.22	168.25	338.64	175.45	368.18	152.23	0.142
DHA RBC (µmol/L)	160.13	116.52	150.06	117.12	175.11	113.39	0.309
EPA RBC (µmol/L)	2.86	2.12	2.64	2.34	2.92	1.72	0.115
LA RBC (µmol/L)	209.49	135.82	193.86	119.7	239.89	140.5	0.029
**Selected daily nutrient intakes evaluated in 24 h dietary recalls**							
Kilocalories	1201.59	494.72	1201.49	440.11	1205.77	580.32	0.889
Protein (g)	66.60	22.11	66.63	23.00	66.48	23.80	0.832
Lipids (g)	44.59	25.08	44.17	22.99	45.67	28.42	0.781
Carbohydrates (g)	135.93	58.15	138.52	51.27	129.93	61.29	0.266
Water (g)	21.96	829.49	2146.43	749.58	2280.80	912.78	0.207
Cholesterol (mg)	260.54	157.94	255.68	129.83	274.79	158.47	0.853
ALA (mg)	77.16	217.21	57.29	196.85	87	272.44	0.280
DHA (mg)	10.63	24	10.16	19.47	10.95	24.74	0.547
EPA (mg)	3.4	5.99	3.09	5.81	4.00	6.20	0.971
LA (mg)	943.08	2306.07	953.40	2072.57	943.08	2334.76	0.527

Legend: PUFA—polyunsaturated fatty acid, IQR—interquartile range (IQR = Q3–Q1), *z*BMI—standardized body mass index (BMI) to account for age and gender, HOMA-IR—homeostatic model assessment for insulin resistance, TC—total cholesterol, TG—triglycerides, HDL—high density cholesterol, g—grams, mg—milligrams, µmol/L—micromoles/liter, AST—aspartate aminotransferase, ALT—alanine aminotransferase, CRP—C reactive protein, HDL chol—high-density lipoprotein cholesterol, ALA—alfa-linolenic acid, ARA—arachidonic acid, DHA—docosahexaenoic acid, EPA—eicosapentaenoic acid, LA—linoleic acid, RBC—red blood cell membrane.

**Table 2:** PUFA levels in RBC membranes grouped by *PEMT* and *MTHFR* genetic variation. The Kruskal–Wallis test was used to analyze differences between genotypes. (Test is significant at *p* value ≤ 0.05).

***PEMT* rs1109859**	**GG, *n* = 12 (6.1%)**		**GA, *n* = 68 (34.7%)**		**AA, *n* = 116 (59.2%)**		***p* Value**
	**Median**	**IQR**	**Median**	**IQR**	**Median**	**IQR**	
ALA RBC (µmol/L)	1.65	1.46	1.54	1.11	1.34	0.99	0.090
ARA RBC (µmol/L)	408.72	112.81	370.48	162.76	366.92	178.73	0.007
DHA RBC (µmol/L)	224.10	151.89	190.15	120.4	144.62	102.81	0.040
EPA RBC (µmol/L)	3.69	2.51	3.09	2.27	2.50	1.83	0.017
LA RBC (µmol/L)	224.02	191.77	243.88	129.83	188.88	116	0.022
***MTHFR* rs4846052**	**TT, *n* = 33 (16.8%)**		**TC, *n* = 97 (49.5%)**		**CC, *n* = 66 (33.7%)**		***p* Value**
	**Median**	**IQR**	**Median**	**IQR**	**Median**	**IQR**	
ALA RBC (µmol/L)	1.54	0.99	1.34	0.92	1.35	1.32	0.061
ARA RBC (µmol/L)	397.38	133.61	337.05	186.2	344.86	148.14	0.006
DHA RBC (µmol/L)	215.69	87.64	142.47	1338.86	153.82	91.28	0.015
EPA RBC (µmol/L)	3.74	2.33	2.67	1.92	2.83	1.77	0.066
LA RBC (µmol/L)	244.93	140.51	199.15	130.24	199.41	134.80	0.044

Legend 2: IQR—interquartile range, ALA—alfa-linolenic acid, ARA—arachidonic acid, DHA—docosahexaenoic acid, EPA—eicosapentaenoic acid, LA—linoleic acid, RBC—red blood cell membrane; *MTHFR*—methylenetetrahydrofolate reductase; *PEMT*—phosphatidylethanolamine N-methyltransferase.

## Appendix A

**Table A1:** Descriptive statistics for fatty acids in plasma and RBC membranes.

Fatty Acids (µmol/L)	Plasma Free		Plasma Conjugated		RBC	
	Median	IQR	Median	IQR	Median	IQR
ALA	3.72	4.27	16.47	15.42	1.41	1.02
ARA	7.15	4.31	375.51	156.61	354.22	168.25
DHA	6.13	4.52	271.70	159.49	160.13	116.52
EPA	0.24	0.14	7.23	5.39	2.86	2.12
LA	137.40	117.75	1208.13	462.86	209.49	135.82

Legend: ALA—alfa-linolenic acid, ARA—arachidonic acid, DHA—docosahexaenoic acid, EPA—eicosapentaenoic acid, LA—linoleic acid, RBC—red blood cell membrane, µmol/L—micromoles/liter, RBC—red blood cells, IQR—interquartile range.

**Table A2:** The frequencies of 55 single nucleotide polymorphisms studied within 196 subjects.

Gene	Rs Chr Position GRCh37	AA/AB/BB	AA%	AB%	BB%
*ABCB4*	rs1149222 chr7:87073775	GG/GT/TT	2.0	28.1	69.9
	rs2071645 chr7:87105276	GG/GC/CC	73.4	25.0	1.6
	rs31672 chr7:87059699	CC/CT/TT	2.6	30.1	67.3
	rs4148811 chr7:87101486	TT/TG/GG	73.5	24.5	2.0
	rs9655950 chr7:87033561	CC/CT/TT	0.5	27.6	71.9
	rs1202283 chr7:87082292	GG/GA/AA	16.0	49.5	34.5
*APOC3*	rs2854117 chr11:116700142	TT/TC/CC	9.7	43.4	46.9
*CHDH*	rs12676 chr3:53857803	AA/AC/CC	9.7	42.9	47.4
	rs2289209 chr3:53852835	CC/CT/TT	89.8	10.2	0.0
	rs4563403 chr3:53850814	CC/CT/TT	78.6	21.4	0.0
	rs4687591 chr3:53864407	AA/AG/GG	86.2	12.8	1.0
	rs6807783 chr3:53859662	GG/GC/CC	65.3	31.1	3.6
	rs7634578 chr3:53876728	CC/CT/TT	98.5	1.5	0.0
	rs881883 chr3:53847805	AA/AG/GG	68.9	28.5	2.6
*CHKB*	rs1557502 chr22:51013998	CC/CT/TT	61.7	32.7	5.6
	rs1557503 chr22:51013072	GG/GA/AA	87.7	11.8	0.5
	rs470117 chr22:51009953	CC/CT/TT	33.2	47.6	19.3
	rs7238 chr22:51007488	AA/AG/GG	80.7	19.3	0.0
*FADS2*	rs2526678chr1161623793	GG/GA/AA	82.1	17.9	0.0
	rs526126chr1161624885	GG/GC/CC	3.1	26.3	70.6
*MTHFD1*	rs10135928 chr14:64866439	TT/TC/CC	95.4	4.6	0.0
*MTHFR*	rs1801133 chr1:11856378	GG/GA/AA	43.4	43.4	13.3
	rs2066471 chr1:11860458	CC/CT/TT	68.4	29.6	2.0
	rs4846048 chr1:11846252	GG/GA/AA	10.7	42.9	46.4
	rs4846052 chr1:11857951	TT/TC/CC	16.9	49.7	33.3
	rs7525338 chr1:11862332	CC/CT/TT	100.0	0.0	0.0
	rs868014 chr1:11849447	AA/AG/GG	0.0	0.0	100.0
*SCD*	rs11557927 chr10:102121816	TT/TG/GG	88.8	10.7	0.5
	rs11599710 chr10:102105788	GG/GA/AA	89.3	10.2	0.5
	rs12247426 chr10:102115327	CC/CG/GG	98.5	1.5	0.0
	rs2167444 chr10:102124744	TT/TA/AA	74.5	22.4	3.1
	rs7849 chr10:102122603	TT/TC/CC	69.6	25.8	4.6
*SLC44A1*	rs10120572chr9108077756	TT/TG/GG	97.4	2.6	0.0
	rs10820799 chr9:108092216	AA/AC/CC	92.3	7.7	0.0
	rs193008 chr9:108042806	TT/TC/CC	83.6	16.4	0.0
	rs328006 chr9:108039808	GG/GC/CC	83.2	16.8	0.0
	rs440290 chr9:107987290	TT/TC/CC	82.7	17.3	0.0
	rs443094 chr9:108016685	GG/GC/CC	84.7	15.3	0.0
	rs7018875 chr9:108077434	CC/CA/AA	95.4	4.6	0.0
*STAT3*	rs9891119 chr17:40507980	AA/AC/CC	41.8	43.9	14.3
*PCYT1A*	rs1580820 chr3:195966258	GG/GA/AA	0.5	20.9	78.6
	rs4898190 chrX:24607933	AA/AC/CC	1.5	6.7	91.8
*PEMT*	rs1109859chr1717424333	GG/GA/AA	6.2	34.9	59.0
	rs12103822 chr17:17418432	CC/CG/GG	99.5	0.5	0.0
	rs16961845 chr17:17432456	CC/CT/TT	83.2	16.3	0.5
	rs4244593 chr17:17420218	TT/TG/GG	20.0	51.8	28.2
	rs4479310 chr17:17405504	CC/CT/TT	12.2	43.9	43.9
	rs7214988 chr17:17491836	CC/CG/GG	83.2	16.3	0.5
	rs7946 chr17:17409560	CC/CT/TT	13.3	44.1	42.6
	rs8068641chr1717480187	AA/AG/GG	78.6	20.9	0.5
	rs936108 chr17:17439793	CC/CT/TT	26.5	52.0	21.4
	rs13342397 chr17:17460926	TT/TC/CC	79.5	19.9	0.6
	rs6502603 chr17:17445680	GG/GT/TT	26.0	52.6	21.4
*PNPLA3*	rs2281135 chr22:44332570 rs738409 chr22:44324727	GG/GA/AA CC/CG/GG	64.3 52.8	33.7 21.1	2.0 26.1

Legend: Gene name as per international nomenclature HUGO Gene Nomenclature Committee (HGNC); Chr—chromosome position reference genome GRCh37; AA—homozygous for one allele; AB—heterozygous; BB—homozygous for the other allele; *ABCB4*—ATP binding cassette subfamily B member 4; *APOC3*—apolipoprotein C3; *CHDH*—choline dehydrogenase; *CHKB*—choline/ethanolamine kinase beta; *FADS2*—fatty acid desaturase 2; *MTHFD1*—methylenetetrahydrofolate dehydrogenase; *MTHFR*—methylenetetrahydrofolate reductase; *SCD*—stearoyl-CoA desaturase; *SLC44A1*—solute carrier family 44 member 1; *STAT3*—signal transducer and activator of transcription 3; *PCYT1A*—phosphate cytidylyltransferase 1, choline, alpha; *PCYT1B*—phosphate cytidylyltransferase 1, choline, beta; *PEMT*—phosphatidylethanolamine N-methyltransferase; *PNPLA3*—patatin-like phospholipase domain containing 3.

**Table A3:** The *p* values for the Mann–Whitney U test used to evaluate differences between PUFA measured in red blood cell membranes in regards to rs1109859 *PEMT*, and rs4846052 *MTHFR* genotypes. Test is significant at *p* ≤ 0.05.

**rs1109859 in *PEMT* gene**					
	**ALA**	**ARA**	**DHA**	**EPA**	**LA**
GG/GA	0.978	0.524	0.201	0.370	0.984
GA/AA	0.059	0.007	0.014	0.029	0.011
GG/AA	0.299	0.058	0.011	0.045	0.202
**rs4846052 in *MTHFR* gene**					
	**ALA**	**ARA**	**DHA**	**EPA**	**LA**
TT/TC	0.020	0.003	0.016	0.027	0.018
TC/CC	0.394	0.624	0.805	0.914	0.734
TT/CC	0.138	0.009	0.004	0.045	0.048

Legend: ALA—alfa-linolenic acid; ARA—arachidonic acid; DHA—docosahexaenoic acid; EPA—eicosapentaenoic acid; LA—linoleic acid; *MTHFR*—methylenetetrahydrofolate reductase; *PEMT*—phosphatidylethanolamine N-methyltransferase.

**Table A4:** Univariate analysis of variance results. Models were created using the PUFA RBC levels as dependent variables; gender, genotypes rs1109859, and rs4846052 as fixed factors, and dietary intakes, hemoglobin, and hematocrit as covariates. Test is significant at *p* ≤ 0.05.

**Dependent Variable: ALA in RBC**					
**Source**	**Type III Sum of Squares**	***df***	**Mean Square**	***F***	**Sig.**
Corrected Model	12.449 ^a^	8	1.556	1.548	0.145
Intercept	6.563	1	6.563	6.528	0.012
Gender	4.011	1	4.011	3.989	0.048
rs1109859 in *PEMT* gene	4.900	2	2.450	2.437	0.091
rs4846052 in *MTHFR* gene	2.513	2	1.256	1.250	0.289
Hemoglobin	0.045	1	0.045	0.045	0.833
Hematocrit	0.356	1	0.356	0.354	0.553
ALA intake	0.275	1	0.275	0.273	0.602
Error	159.858	159	1.005		
Total	656.808	168			
Corrected Total	172.307	167			
a. *R* Squared = 0.072 (Adjusted *R* Squared = 0.026)					
**Dependent Variable: ARA in RBC**					
**Source**	**Type III Sum of Squares**	***df***	**Mean Square**	***F***	**Sig.**
Corrected Model	552433.852 ^a^	7	78919.122	3.657	0.001
Intercept	284923.202	1	284923.202	13.204	0.000
Gender	38020.108	1	38020.108	1.762	0.186
rs1109859 in *PEMT* gene	290956.552	2	145478.276	6.742	0.001
rs4846052 in *MTHFR* gene	92203.136	2	46101.568	2.136	0.121
Hemoglobin	69691.519	1	69691.519	3.230	0.074
Hematocrit	95978.498	1	95978.498	4.448	0.036
Error	3927257.216	182	21578.336		
Total	30801660.185	190			
Corrected Total	4479691.068	189			
a. *R* Squared = 0.123 (Adjusted *R* Squared = 0.090)					
**Dependent Variable: DHA in RBC**					
**Source**	**Type III Sum of Squares**	***df***	**Mean Square**	***F***	**Sig.**
Corrected Model	111644.265 ^a^	8	13955.533	1.831	0.075
Intercept	44569.962	1	44569.962	5.847	0.017
Gender	6232.763	1	6232.763	0.818	0.367
rs1109859 in *PEMT* gene	69225.385	2	34612.692	4.541	0.012
rs4846052 in *MTHFR* gene	33145.490	2	16572.745	2.174	0.117
Hemoglobin	2690.465	1	2690.465	0.353	0.553
Hematocrit	3791.682	1	3791.682	0.497	0.482
DHA intake	943.244	1	943.244	0.124	0.725
Error	1212070.616	159	7623.086		
Total	7032444.484	168			
Corrected Total	1323714.880	167			
a. *R* Squared = 0.084 (Adjusted *R* Squared = 0.038)					
**Dependent Variable: EPA in RBC**					
**Source**	**Type III Sum of Squares**	***df***	**Mean Square**	***F***	**Sig.**
Corrected Model	33.140 ^a^	8	4.143	0.853	0.558
Intercept	19.043	1	19.043	3.921	0.049
Gender	2.858	1	2.858	0.588	0.444
rs1109859 in *PEMT* gene	21.406	2	10.703	2.204	0.114
rs4846052 in *MTHFR* gene	5.710	2	2.855	0.588	0.557
Hemoglobin	0.758	1	0.758	0.156	0.693
Hematocrit	1.599	1	1.599	0.329	0.567
EPA intake	1.324	1	1.324	0.272	0.602
Error	772.318	159	4.857		
Total	2855.067	168			
Corrected Total	805.458	167			
a. *R* Squared = 0.041 (Adjusted *R* Squared = −0.007)					
**Dependent Variable: LA in RBC**					
**Source**	**Type III Sum of Squares**	***df***	**Mean Square**	***F***	**Sig.**
Corrected Model	220014.263 ^a^	8	27501.783	2.829	0.006
Intercept	142585.361	1	142585.361	14.665	0.000
Gender	86981.037	1	86981.037	8.946	0.003
rs1109859 in *PEMT* gene	64090.372	2	32045.186	3.296	0.040
rs4846052 in *MTHFR* gene	19491.620	2	9745.810	1.002	0.369
Hemoglobin	13805.541	1	13805.541	1.420	0.235
Hematocrit	29920.071	1	29920.071	3.077	0.081
LA intake	21692.633	1	21692.633	2.231	0.137
Error	1545925.144	159	9722.800		
Total	10938772.094	168			
Corrected Total	1765939.407	167			
a. *R* Squared = 0.125 (Adjusted *R* Squared = 0.081)					

Legend: DHA—docosahexaenoic acid, EPA—eicosapentaenoic acid, LA—linoleic acid, RBC—red blood cells, *MTHFR*—methylenetetrahydrofolate reductase, *PEMT*—phosphatidylethanolamine *N* -methyltransferase.
